# Adaptive-Compression Based Congestion Control Technique for Wireless Sensor Networks

**DOI:** 10.3390/s100402919

**Published:** 2010-03-29

**Authors:** Joa-Hyoung Lee, In-Bum Jung

**Affiliations:** Department of Computer Science and Engineering, Kangwon National University, Chuncheon, Gangwondo, 200-701, Korea; E-Mail: jinnie4u@kangwon.ac.kr

**Keywords:** wireless sensor network, congestion, compression, queue control

## Abstract

Congestion in a wireless sensor network causes an increase in the amount of data loss and delays in data transmission. In this paper, we propose a new congestion control technique (ACT, Adaptive Compression-based congestion control Technique) based on an adaptive compression scheme for packet reduction in case of congestion. The compression techniques used in the ACT are Discrete Wavelet Transform (DWT), Adaptive Differential Pulse Code Modulation (ADPCM), and Run-Length Coding (RLC). The ACT first transforms the data from the time domain to the frequency domain, reduces the range of data by using ADPCM, and then reduces the number of packets with the help of RLC before transferring the data to the source node. It introduces the DWT for priority-based congestion control because the DWT classifies the data into four groups with different frequencies. The ACT assigns priorities to these data groups in an inverse proportion to the respective frequencies of the data groups and defines the quantization step size of ADPCM in an inverse proportion to the priorities. RLC generates a smaller number of packets for a data group with a low priority. In the relaying node, the ACT reduces the amount of packets by increasing the quantization step size of ADPCM in case of congestion. Moreover, in order to facilitate the back pressure, the queue is controlled adaptively according to the congestion state. We experimentally demonstrate that the ACT increases the network efficiency and guarantees fairness to sensor nodes, as compared with the existing methods. Moreover, it exhibits a very high ratio of the available data in the sink.

## 1. Introduction

Recent advances in MEMS (micro electro mechanical systems) and microprocessor and wireless communication technologies have enabled the deployment of large-scale sensor networks, where thousands or even tens of thousands of small sensors are distributed over a vast area in order to collect sensing data. Sensor networks have attracted significant attention as important infrastructure for data collection in pervasive computing environments. In this field, wireless sensor networks play a special role in home automation, environmental monitoring, military, health, and other applications [1–9].

Some of these applications require sensor nodes to send data continuously, whereas in some applications, sensor nodes should send data only when a specified event or phenomenon occurs. With fast continuous transmission, the variation of the phenomenon in the environment can be monitored precisely; however, the dissipation of energy, which is a very critical resource, can drastically increase. The energy consumption can be reduced by sending only the information of an event occurrence, but the variation in the phenomenon cannot be monitored in detail. For energy efficiency and detailed monitoring, sensor nodes should control the data transmission interval in an inverse proportion to the variation in the phenomenon. Therefore, the transfer rate could be also varied according to the event occurrence. However, variable transfer rates might cause network congestion due to concentrated packets in case of a concurrent occurrence of multiple events. During congestion, sensor nodes usually drop the overflowed packets; however, packet drops lead to data loss and unnecessary energy dissipation. Therefore, routing protocols should efficiently control the network congestion [9–17].

**Figure 1.**
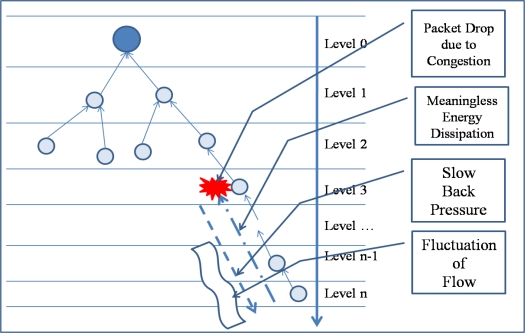
Congestion control by back-pressure scheme.

Conventional congestion control protocols usually use a back-pressure scheme, which reduces congestion by reducing the transfer rate of child nodes of a congested node and by decreasing the packet generation rate of a sensor node that causes heavy traffic. However, packet drops might still occur because the back pressure is propagated slowly due to the collision among sensor nodes in case of serious congestion. Slow propagation of the back pressure may also cause fluctuation in packet flows because network state changes frequently with the back pressure. Moreover, a decrease in the packet generation rate leads to a reduction in the fidelity of an event because heavy traffic is generated by a sensor node that detects the specified event. Figure 1 shows the problem of congestion control with the back pressure [17–35].

In order to reduce congestion, the routing protocol should reduce the number of packets in the network; however, simply dropping overflowed packets will reduce the data fidelity and increase the energy dissipation. In this paper, we propose a new congestion control technique (ACT, Adaptive Compression-based congestion control Technique) based on an adaptive compression scheme for packet reduction in case of congestion. Compression techniques used in the ACT are Discrete Wavelet Transform (DWT), Adaptive Differential Pulse Code Modulation (ADPCM), and Run-Length Coding (RLC). The ACT first transforms the data from the time domain to the frequency domain, reduces the range of the data with the help of ADPCM, and then reduces the number of packets by means of RLC before transferring the data to the source node. It then introduces the DWT for priority-based congestion control because the DWT classifies the data into four groups with different frequencies. Thereafter, it assigns priorities to these data groups in an inverse proportion to the respective frequencies of the data groups and defines the quantization step size of ADPCM in an inverse proportion to the priorities. RLC generates a less number of packets for a packet with a low priority. In the relaying node, the ACT reduces the number of packets by increasing the quantization step size of ADPCM in case of congestion. Moreover, in order to facilitate the back pressure, the queue is controlled adaptively according to the congestion state. In the next section, we review related studies. Section 3 describes the ACT, and Section 4 provides a performance evaluation. We conclude the paper in Section 5.

## 2. Related Studies

A number of previous studies have addressed the topic of congestion control in wireless sensor networks. Most existing congestion control protocols usually adjusts traffic rate at source nodes or intermediate nodes. This approach is helpful to save network resources and more feasible and efficient. According to the control behavior, there are two general methods for traffic control in WSNs: end-to-end and hop-by hop. The end-to-end control can impose exact rate adjustment at each source node and simplify the design at intermediate nodes; however, it results in slow response and relies highly on the round-trip time (RTT). In contrast, the hop-by-hop congestion control has faster response.

CODA [6] (congestion detection and avoidance) is a congestion mitigation strategy that uses both the buffer occupancy and channel load for measuring congestion levels in a network. It uses two strategies for handling both persistent and transient congestions. CODA performs rate adjustment through a traditional TCP-like AIMD (additive increase multiplicative decrease) mechanism and thus often leads to the occurrence of packet loss. Further, Fusion detects congestion by measuring the queue length. It controls congestion by combining three techniques: hop-by-hop flow control, source rate limiting, and prioritized MAC. Fusion claims to achieve good throughput and fairness at a high offered load [20,24].

In [14], a congestion control technique that employs the packet service time to infer the available service rate and therefore detects congestion in each intermediate sensor node was proposed. It controls congestion in a hop-by-hop manner and uses exact rate adjustment based on its available service rate and number of child nodes. However, it cannot utilize the available link capacity efficiently when some nodes are in the sleep state. Siphon [35] is another congestion mitigation scheme that detects congestion using the queue length. However, instead of using any rate adjustment technique, it employs traffic redirection in order to mitigate congestion. Very recently, node-priority-based congestion control mechanisms have been proposed [15,16,27,33]. The Priority-based Congestion Control Protocol (PCCP) [15] and Queue based Congestion Control Protocol with Priority Support (QCCP-PS) [16] control the congestion with the packet priority based on the node priority for a WSN. PCCP introduced node priority index to reflect the importance of each sensor node. Based on the congestion degree and node priority index, PCCP utilizes a cross-layer optimization and imposes a hop-by-hop approach to control congestion. QCCP-PS improved the PCCP by controlling the queue more finely. However, it does not have any mechanism for handling prioritized heterogeneous traffic in the network.

Recently, there have been a number of research works attempting to increase the sensor node data transmission throughput, packet delivery ratio and data security via multipath routing [17,25,30,31]. In [17] its authors proposed a source data packet loading scheme over multiple paths and then presented congestion detection and control algorithms suited for multipath data forwarding. At first a source node starts to send data packets over two different paths at a predefined rate. The congestion detection algorithm at each intermediate node is invoked at reception of every data packet. If congestion is detected, a congestion notification packet is sent back to the source and then the source invokes the proposed congestion control algorithm to readjust the packet loading rate.

Congestion-aware and Rate-controlled Reliable Transport (CRRT) [19] uses efficient MAC retransmission to increase one-hop reliability and end-to-end retransmission for loss recovery. CRRT centrally assigns the rate to the source based on the rate assignment policy of application. The Cross-Layer Active Predictive Congestion Control (CL-APCC) scheme [21] for improving the performance of networks applied Queuing theory to analyze data flows of a single-node according to its memory status, combined with the analysis of the average occupied memory size of local networks. It also analyzes the current data change trends of local networks to forecast and actively adjust the sending rate of the node in the next period. In order to ensure the fairness and timeliness of the network, the IEEE 802.11 protocol is revised based on waiting time, the number of the node’s neighbors and the original priority of data packets, which dynamically adjusts the sending priority of the node.

Decentralized, Predictive Congestion Control [22] (DPCC) for wireless sensor networks (WSN) consists of an adaptive flow and adaptive back-off interval selection schemes that work in concert with energy efficient, distributed power control (DPC). The DPCC detects the onset of congestion using queue utilization and the embedded channel estimator algorithm in DPC that predicts the channel quality. Then, an adaptive flow control scheme selects suitable rate which is enforced by the newly proposed adaptive back-off interval selection scheme. Auction-based mechanism for providing efficient and localized WSN transient congestion management based on applications’ priorities and the utility of sensed information was proposed in [23]. An essential component of this mechanism is a partially user-defined information utility metric that jointly represents two components. One is the objective quality of information (QoI), at which targets are tracked. The other is the subjective priority of each application defined by the user to reflect the value of knowing the precise position of a target. The combined metric is defined as the product of the tracking imprecision at the source sensor, the delay with which target updates are received, and the application’s priority. This relation is clear for the tracking imprecision at the source sensor or delay of the packet carrying the measurements. Inclusion of priority in the metric enables us to differentiate between utility losses of different missions. DiffQ [29] provide the practical adaptation and implementation of differential backlog that involves a cross layer optimization of both congestion control and MAC scheduling in real multi-hop wireless. One of the main goals of DiffQ is to measure the performance difference between differential back-log and the other existing practical, but ad hoc, solutions. DiffQ adapts the original solution for practical implementation using several heuristics for scheduling and routing [32].

## 3. ACT

### 3.1. Problem

There are mainly two types of congestion in WSNs. The first type is the node-level congestion which occurs due to queue overflow inside the node. Queue overflow might lead to packet drop and this leads to retransmission if required and therefore consumes additional energy. Wireless channels are shared by several nodes using Carrier Sense Multiple Access (CSMA)-like protocols and thus collisions among sensor nodes can occur when multiple sensor nodes try to occupy the channel concurrently. This is the second type of congestion-link-level congestion. Link-level congestion increases the packet service time and decreases the link utilization. Both the node level and the link-level congestions have direct impact on energy efficiency and Quality of Data (QoD). Therefore congestion must be efficiently controlled. Congestion control protocol efficiency depends on how much it can achieve the following objectives: first, energy-efficiency should be improved in order to extend system lifetime. Therefore congestion control protocols need to avoid or reduce packet loss due to buffer overflow, and remain lower control overhead that consumes less energy; second, it is also necessary to support traditional QoS metrics such as packet loss ratio, packet delay, and throughput; third, fairness needs to be guaranteed so that each node can achieve fair throughput [15].

The main purpose of the proposed ACT scheme is to guarantee a high quality of data by reducing dropped packets due to the congestion. First ACT reduces the amount of generated packets on the source node with compression scheme-DWT, ADPCM, and RLC. Second ACT reduces the amount of transmitting rate on the relaying node with compression scheme under the congestion by adjusting the quantization step on the ADPCM. Third ACT assigns the priority based on the result of DWT to guarantee the reconstruction of data with packet loss. Last, for the fast propagation of congestion notification, the queue is operated adaptively according to the congestion state and queue state.

### 3.2. Network and Node Model

We consider a densely deployed wireless sensor network such that each sensor node has one or more sensing devices. A sensor node frequently transfers the sensed data to a sink node or gateway. Sensor nodes should control the data transmission interval in an inverse proportion to the variation in a phenomenon. A tree-based routing protocol is used to construct the path from sensor nodes to the sink node. The path to the sink node can be changed whenever the link states to the upper nodes are unreliable due to node failure or obstacles. The lower nodes in a routing tree have to compete with other nodes for an upper node in a routing tree. The errors and losses that occur in the air are corrected by an error-correcting code such as FEC or ACK, which are based on a retransmission technique.

**Figure 2.**
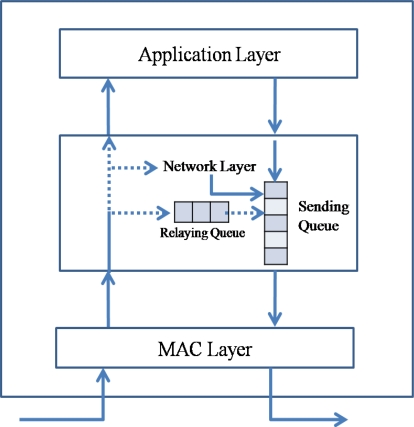
Packet flow in a sensor node.

Figure 2 presents the queuing model in a sensor node. A sensor node has two queues, a relaying queue for transit traffic and a sending queue for transferring data. The packets generated by the sensor node itself are inserted directly into the sending queue. A sensor node could generate a data packet for sensed data in an application layer or a routing packet for routing information in a network layer. Therefore, the packets received from the network also can be one of two packet types, a data packet and a routing packet. If the destination address of a data packet is the receiving node, the packet is transferred to an upper application layer. If the destination address is not the receiving node, the packet is inserted into the relaying queue and then into the sending queue. A routing packet is used by the routing protocol in a network layer.

A sensor network is a type of *ad-hoc* network that is deployed without any infrastructure. Therefore, a sensor node should not only act as a data source but also as a relaying node. Packets in the sending queue of a sensor node can be classified into a transit packet received from another node and a source packet generated by the sensor node itself. The routing protocol should reduce the insertion rate of not only the transit packet but also the source packet. Reducing the transit traffic is called as the back pressure, and reducing the source traffic is referred to as the up pressure.

### 3.3. Compression

#### 3.3.1. Compression Technique

The ACT uses three types of compression techniques, namely, DWT, ADPCM, and RLC. DWT and RLC are usually used in image compression such as JPEG2000, and ADPCM is used in audio recording [36–40].

Wavelet transform exploits both the spatial and frequency correlation of data by dilations and translations of mother wavelet on the input data. It supports the multi resolution analysis of data, i.e., it can be applied to different scales according to the details required, which allows progressive transmission and zooming of the data without the need of extra storage. Another encouraging feature of wavelet transform is its symmetric nature that is both the forward and the inverse transform has the same complexity, building fast compression and decompression routines. The Discrete Wavelet Transform (DWT), which is based on sub-band coding is found to yield a fast computation of the Wavelet Transform. It is easy to implement and reduces the computation time and resources required. A time-scale representation of the digital signal is obtained using digital filtering techniques. The signal to be analyzed is passed through filters with different cutoff frequencies at different scales. The DWT is computed by successive low-pass and high-pass filtering of the discrete time-domain signal. This is called the Mallat algorithm or Mallat-tree decomposition. Its significance is in the manner it connects the continuous-time multi resolution to discrete-time filters. At each level, the high pass filter produces detail information, while the low pass filter associated with scaling function produces coarse approximations. The Wavelet Transform at high frequencies gives good time resolution and poor frequency resolution, while at low frequency the Wavelet Transform gives good frequency resolution and poor time resolution. Its characteristics well suited for data compression include the ability to take into account of very good energy compaction capabilities, robustness under transmission, high compression ratio etc. Wavelet transform divides the information of data into approximation and detail sub-signals. The approximation sub-signal shows the general trend of data values and other three detail sub-signals show the vertical, horizontal and diagonal details or changes in the data.

Adaptive DPCM (ADPCM) is a variant of Differential Pulse-Code Modulation (DPCM) that varies the size of the quantization step, to allow further reduction of the required bandwidth for a given signal-to-noise ratio. DPCM or differential pulse-code modulation is a signal encoder that uses the baseline of PCM but adds some functionalities based on the prediction of the samples of the signal. The input can be an analog signal or a digital signal. A quantizer is used to reduce the number of bits needed to store the transformed coefficients by reducing the precision of those values. As it is a many-to-one mapping, it is a lossy process and is the main source of compression in an encoder. Quantization can be performed on each individual coefficient, which is called Scalar Quantization (SQ). Quantization can also be applied on a group of coefficients together known as Vector Quantization (VQ). Both uniform and non-uniform quantizers can be used depending on the problems.

An entropy encoder supplementarily compresses the quantized values losslessly to provide a better overall compression. It uses a model to perfectly determine the probabilities for each quantized value and produces an appropriate code based on these probabilities so that the resultant output code stream is smaller than the input stream. The most commonly used entropy encoders are the Huffman encoder and the arithmetic encoder, although for applications requiring fast execution, simple Run Length Coding (RLC) is very effective. It is important to note that a properly designed quantizer and entropy encoder are absolutely necessary along with optimum signal transformation to get the best possible compression.

These compression techniques are usually used in compression of images or audio which are continuous data and have similar data values. We use these compression techniques because the sensed data are continuous and have similar values. DWT, ADPCM, and DWT can drastically reduce the number of packets required by the sensed data.

**Figure 3.**
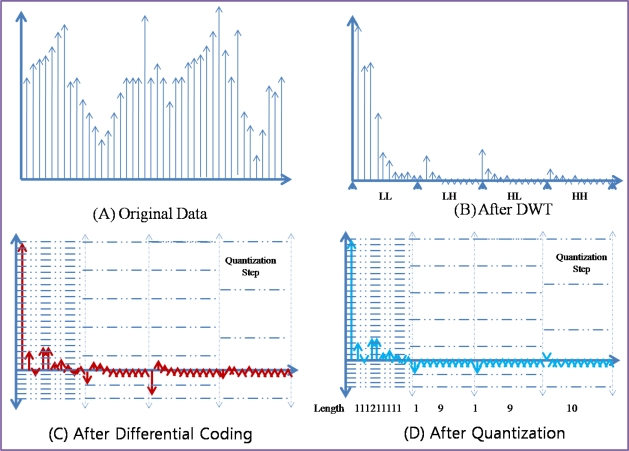
Example of Compression Techniques in ACT.

Figure 3 shows an example of compression techniques in ACT. With original data [Figure 3-(A)] which was sensed by sensor node, DWT transforms the data from the time sequence domain to the frequency domain [Figure 3-(B)]. With DWT, most of energy in the data is concentrated to the LL sub-band and other sub-bands have less energy than that of LL sub-band. Differential coding is applied to the result values of DWT [Figure 3-(C)]. With differential coding, the values become small and the range of value is reduced. Each sub-band has different rage of values and thus different quantization step size is applied to each sub-band. As a consequence, with adaptive quantization, most of values in high frequency become 0. After quantization, RLC produces a very small amount of bit stream [Figure 3-(D)].

#### 3.3.2. Applicability

TinyOS Tools for Wavelet Decomposition on motes [41] provides a modular library of components to facilitate the construction of application-specific progressive transmission or lossy compression solutions over WSN devices, or sensor motes in wireless sensor network. This project provides a multi-resolution data transmission library in order to reduce latency/bandwidth/energy of data transmission after vibration events. Efficient Integer-Integer Wavelet Lifting Transform is a wavelet lifting transform that uses only integer operations and can decompose data in real-time for data rates as high as 250 Hz. The filter was chosen after evaluating the performance over building vibration signals that were obtained from tests. Threshold builds a histogram of the signal to determine ideal threshold for signal de-noising and compression. Quantization is a uniform quantizer that can be used to reduce the resolution of data depending on the range of the signal and number of bits allocated to each sample. Run-Length Encoding is an encoder that takes a thresholded/quantized wavelet decomposed stream, compresses it and packs it into a bit-stream. Bit-Stream is an embedded bit-stream that can be used to pack samples depending on resolution of each sample. EEPROM Circular Buffer Manager partitions the EEPROM into different circular buffers that can be used for event storage. These modules can be used independently. One application may decide to use solely quantization followed by packing data in a bit-stream.

Distributed Wavelet Transform for Wireless Sensor Networks [42] implements data compression for wireless sensor networks by using a distributed wavelet transform. This compression system lowers the number of devices which need to transmit data to a base station, and thus reduces power consumption and increases the average lifetime of the network. The source code running on the mote, was written in nesC for TinyOS [9] and tested on Crossbow’s MicaZ platform [43]. The first was a suite of networking components. As the focus of this project is not on routing protocols, we opted for simplicity by using basic broadcast and unicast protocols. The broadcast protocol waits for packets with a sequence number larger than the last sequence number received, and then repeats those new packets a set number of times. The unicast protocol uses a static routing table to determine the next hop for a given packet. Above these protocols, a multi-packet fragmentation and reassembly service were built. It allows for bidirectional communication of any data size. TinyOS 1.x uses a fixed data length of 29 bytes, so expanding beyond this limit in a reusable manner simplifies code significantly. By using small descriptor records, this system can even rebuild data structures that use pointers or variable-length arrays. These techniques were used to send the initial wavelet transform parameters to the motes and also to request statistical data from the motes about network traffic. The distributed wavelet transform is the core application running on top of these and other standard TinyOS system services. To ensure that each mote runs each scale of the transform at the same time, a finite state machine with fixed-length delays between each state is used. To achieve data compression, each mote compares its value against a list of target values from the sink, which are arranged in decreasing order. Each mote compares its results from the current round of the wavelet transform with the target values. The first target value that is less than the result value determines the transmission band. Each mote assigned to a specific band transmits its data back to the sink at roughly the same time. When the sink determines it has received enough values to reconstruct a good approximation of the original data, it broadcasts a stop message to prevent further bands from being transmitted. This message also includes updated target values that will be used during the next transform round.

#### 3.3.3. Compression in ACT

**Figure 4.**
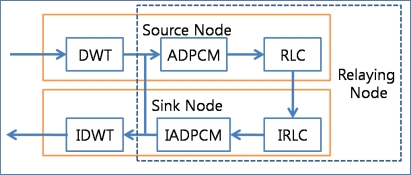
Data compression procedure.

Figure 4 shows the usage of data compression techniques in the ACT. The ACT first transforms the data from the time domain to the frequency domain by using the DWT, reduces the range of the data with the help of ADPCM, and then reduces the number of packets by employing RLC before transfer of data in source node. Then, it introduces the DWT for priority-based congestion control because DWT classifies the data into four groups with different frequencies. Subsequently, it assigns priorities to these data groups in an inverse proportion to the respective frequencies of the data groups and defines the quantization step size of ADPCM in an inverse proportion to the priorities. RLC generates a less number of packets for a packet with a low priority. In the relaying node, the ACT reduces the number of packets by increasing the quantization step size of ADPCM in case of congestion. The destination node (usually a sink node) reverses the compression procedure. A sink node should apply Inverse Run-Length Coding (IRLC), Inverse Adaptive Differential Pulse Code Modulation (IADPCM), and then Inverse Discrete Wavelet Transform (IDWT).

**Figure 5.**
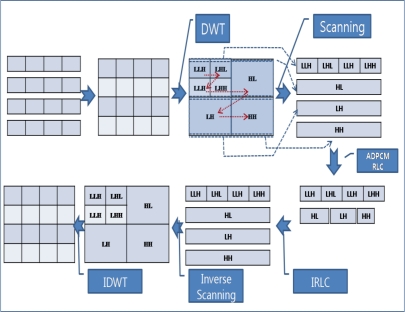
Data compression example.

Figure 5 shows an example of compression in the ACT. First, several packets are grouped into a square array with the same width and height. Then, the DWT transforms the data from the time domain to the frequency domain, and the array can be divided into four sections, LL (low and low frequency) on the upper left side, LH (low and high frequency) on the upper right side, HL (high and low frequency) on the lower left side, and HH (high and high frequency) on the lower right side. If the DWT is applied one more time, the LL section is divided into four sections, as shown in the Figure 5. The LL section presents the shape of data in a reduced resolution, and the original data can be reconstructed without a high frequency. The ACT assigns a high priority to the section with a low frequency. The data transformed by the DWT should be scanned from a low frequency to a high frequency, as shown in the Figure. After scanning, ADPCM and RLC are applied, reducing the number of packets. A sink node applies the following reversed procedures: IRLC, inverse scanning, and IDWT.

### 3.4. APC and ARC

**Figure 6.**
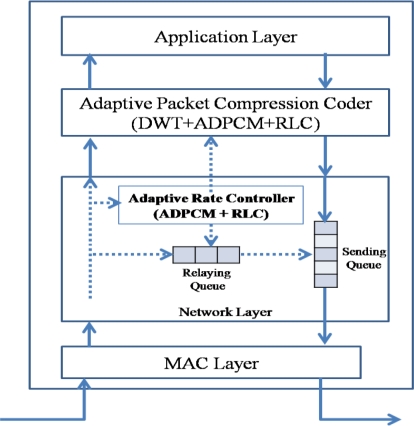
APC and ARC.

The ACT consists of an APC (adaptive packet compression coder) and an ARC (adaptive rate controller), as shown in Figure 6.

The ACP applies the compression techniques (DWT, ADPCM, and RLC) to the source packet generated by the application layer; in case of a sink node, it applies the inverse compression techniques (IDWT, IADPCM, and IRLC). The ARC controls the packet rate by reducing the number of transit packets in the relaying queue with the help of ADPCM and RLE. It is a reduced version of the APC without DWT. One may implement only the APC and then use only ADPCM and RLC for the ARC. If the APC and ARC are implemented separately, ADPCM and RLC component (or procedure) can be shared between the APC and the ARC.

#### 3.4.1. APC

**Figure 7.**
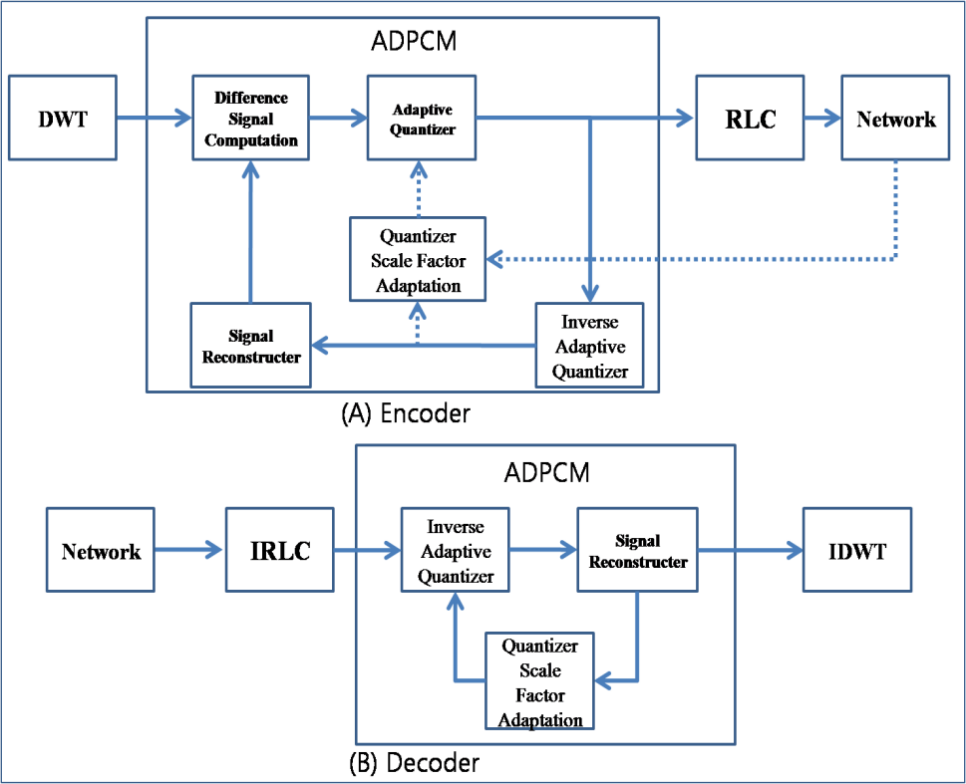
Block schematic of APC.

Figure 7 shows the block schematic of the APC. The DWT and RLC are similar to the ones conventionally used, and ADPCM is a reduced version used for sensed data compression. The encoder of the ADPCM consists of a difference signal computation, adaptive quantizer, inverse adaptive quantizer, quantizer scale factor adaptation, and signal reconstructor. The difference signal computer subtracts the reconstructed signal from the original data. The subtraction of two successive data packets that have similar values reduces the range of data values. The range of values is reduced again by the adaptive quantizer. The quantized data is encoded by RLC and then inserted into the network layer. The quantized data are also inversely quantized by an inverse adaptive quantizer and reconstructed into the signal by the signal reconstructor. The number of generated packets is varied by the adaptive quantizer, which is controlled by the quantizer scale factor adaptation. The quantizer scale factor adapter increases the quantizer step size in proportion to the strength of congestion. Further, the decoder of the ADPCM consists of an inverse adaptive quantizer, signal reconstructor, and quantizer scale factor adapter.

#### 3.4.2. ARC

**Figure 8.**
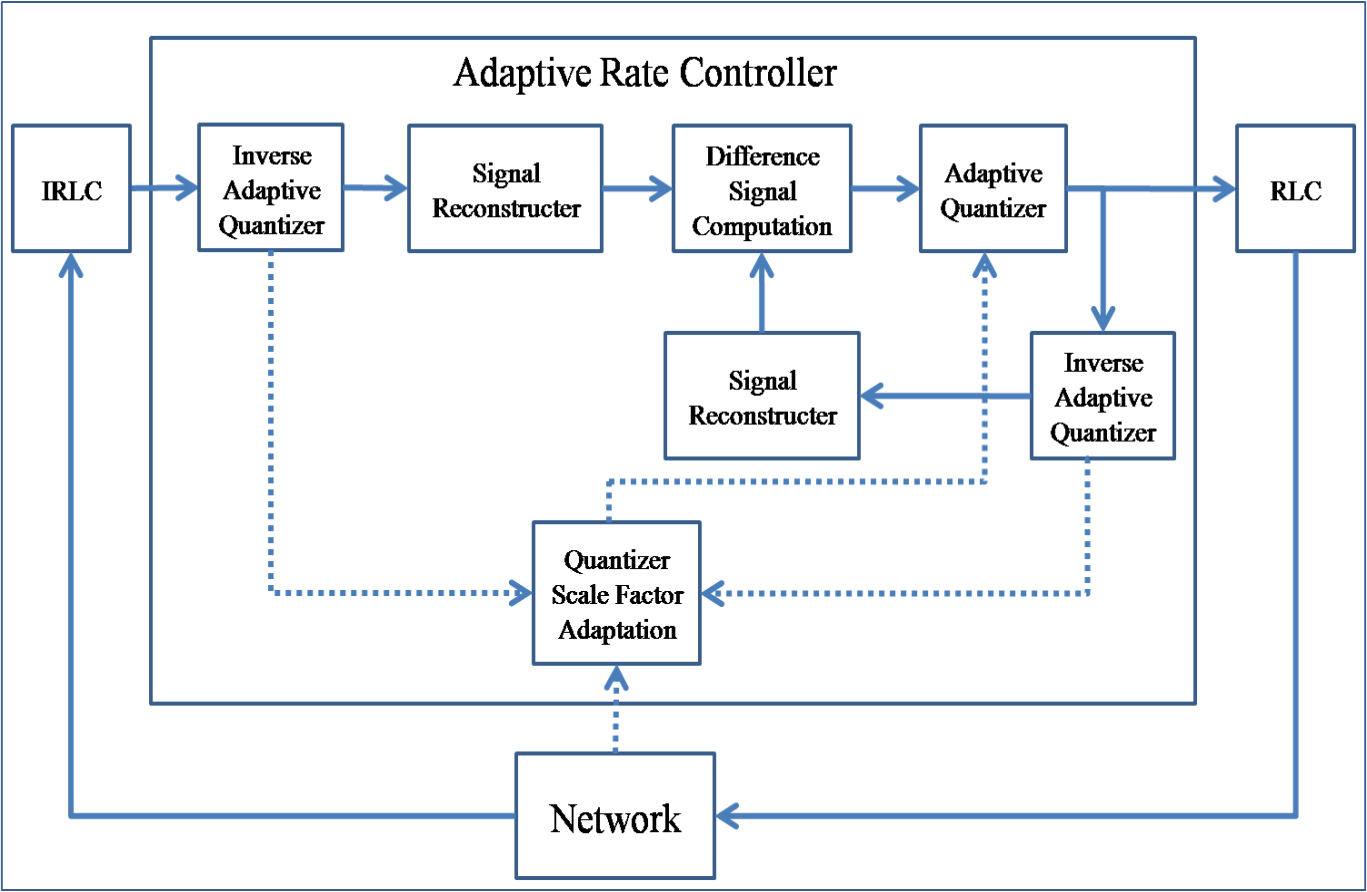
Block schematic of ARC.

The ARC controls the output packet rate by re-encoding the transit packets in the relaying queue. It consists of RLC, IRLC, ADPCM, and IADPCM units. Figure 8 shows its block schematic. Transit packets are in a compressed state after RLC is performed, and therefore, the ARC first decodes them with the help of IRLC. Then, the decompressed transit packets are reconstructed by IADPCM and compressed again with a different quantizer step size, which is affected by the congestion state.

#### 3.4.3. Operation

**Figure 9.**
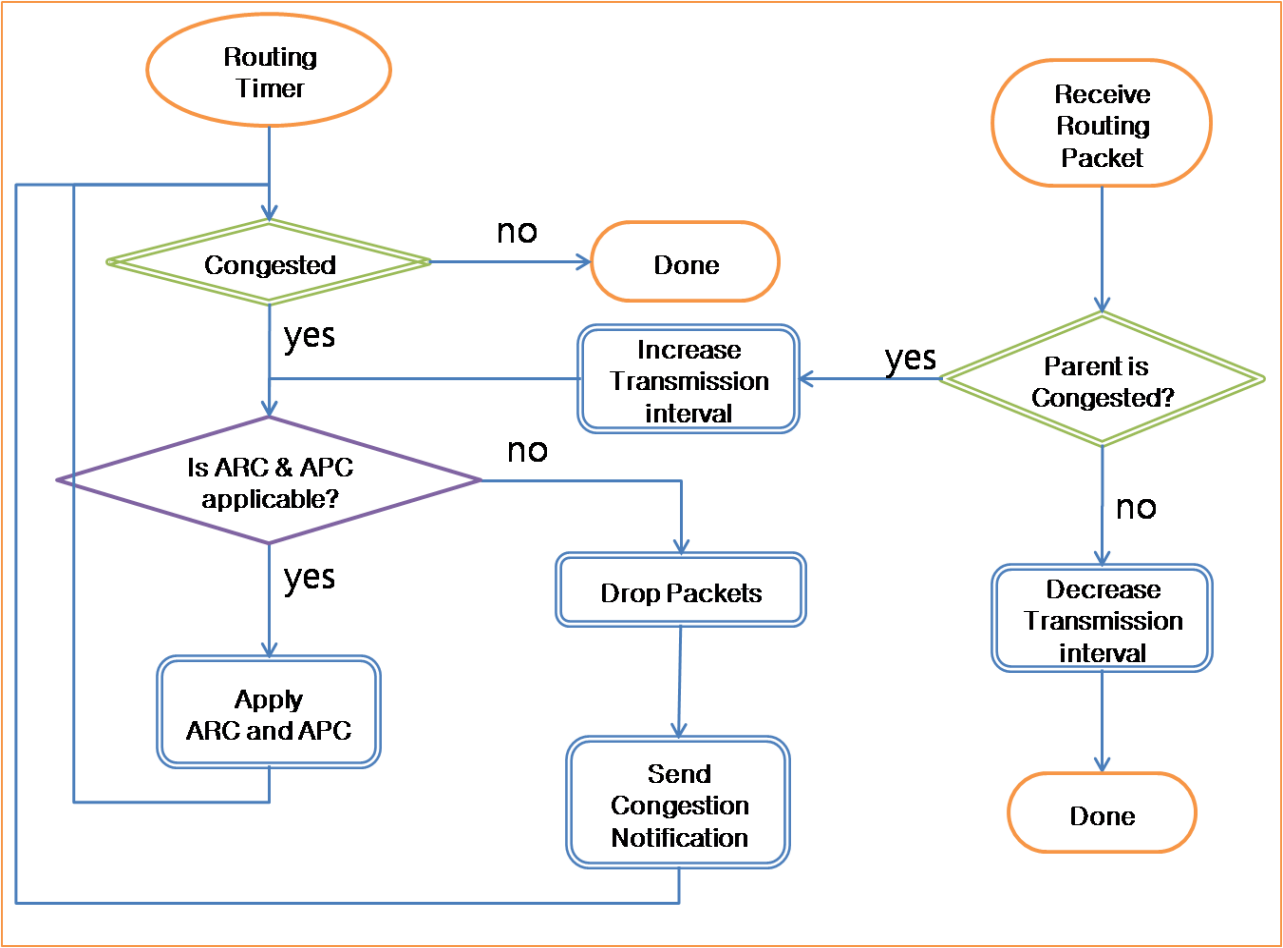
Operation of ACT.

Figure 9 presents the operation of the ACT. The ACT checks the queue state periodically with the routing timer. If the queue is congested with packets, then the ACT checks whether the ARC and APC are applicable or not. If the ARC and APC are applicable, the ACT applies the APC for source packets and the ARC for transit packets. If the congestion persists, the quantization step size is increased drastically. If the quantization step size reaches the limit and the queue is still congested, the ACT starts to drop packets with a low priority in the queue and send routing packets with congestion notification. If a sensor node receives a routing packet with congestion notification from the parent node, the child sensor node increases the transmission interval of packets in the queue and checks whether the ARC and APC are applicable. If the child node faces congestion similar to the parent node, it propagates the congestion notification to its child nodes.

### 3.5. Adaptive Queue Operation in Congestion

**Figure 10.**
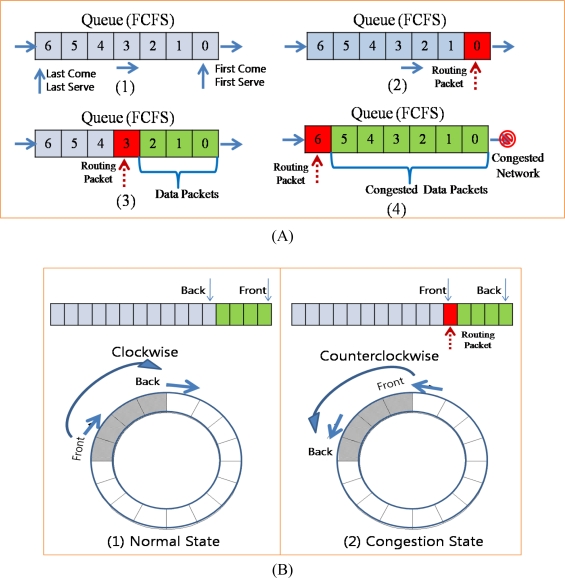
Queue operation in congestion.

The queue in a network layer works in the First Come First Serve (FCFS) mode [Figure 10-(A)-(1)]. If there is no packet in the sending queue, the packet is served immediately [Figure 10-(A)-(2)]. If some packets are already in the queue, the packet that was inserted last has to wait for the previous packets to be served [Figure 10-(A)-(3)]. If there is no congestion, packets will be served in the order in which they were inserted. In contrast, if packets are congested in the queue, they must wait until the congestion is reduced [Figure 10-(A)-(4)]. Data packets may be delayed for some time or dropped in congestion. However, routing packets should not be delayed or dropped because during congestion, they must be propagated fast so as to reduce the traffic from child nodes. In a conventional congestion control protocol, the back pressure is propagated slowly because routing packets are considerably delayed due to the congested queue. In order to address this problem, the ACT controls the direction for fast propagation of congestion notification. During congestion, if the queue is served in the clockwise direction and a routing packet is inserted at the end of the queue (pointed by the back pointer), the ACT changes the direction to counterclockwise, and thus, the back and front pointers are also changed. Therefore, the routing packet inserted at the last position in the queue becomes the first packet in the queue and is served first. In this case, if the first packet in the queue is a data packet, it cannot be served until a reply packet is received from the parent node. However, a routing is broadcasted to the child nodes and therefore can be served directly even if the parent is congested. As a consequence, a routing packet with congestion notification can be propagated fast to the child nodes.

### 3.6. Compression and Congestion

These compression techniques are usually used in compression of images or audio which are continuous data and have similar data values. We use these compression techniques because the sensed data are continuous and have similar values. DWT, ADPCM, and DWT can drastically reduce the number of packets required by the sensed data. Moreover, in case of congestion, the probability of a packet drop can be reduced by applying ADPCM with a big quantization step size, which reduces the amount of output data from RLC and thus the number of required packets. Dropping a packet causes the loss of the entire data contained in the dropped packet and thus reduces the amount of data, while increasing the quantization step size reduces the fineness (fidelity) of the data.

**Figure 11.**
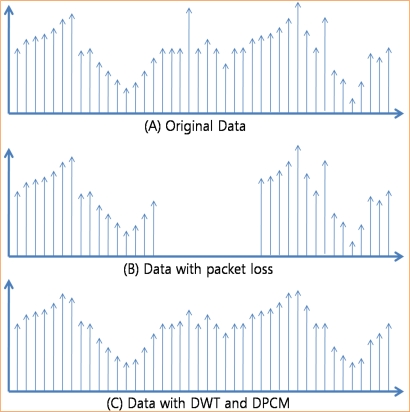
Packet loss and compression.

Figure 11 shows the different of packet dropping and data compression with network congestion. We assume that values of the original data in Figure 11–(A) which were sensed by the sensor device on the source node are continuous stream of integer number and are similar with each other. However sometimes values might be different with other due to the events or variations in environment. With packet dropping technique only, the whole data on the dropped packets are lost and cannot be available to users as shown in Figure 11-(B). To recover the lost data, many congestion control protocol uses end-to-end retransmission, which requires energy consumption. On the other hand, with data compression – DWT and DPCM – packets with lower priority (with high frequency) are dropped first in case of congestion, and thus the sink node can reconstruct the original data with higher priority data (with low frequency) as shown in Figure 11-(C). Dropping the high frequency data removes the spike data which is very different value with other data and thus the reconstructed data are smoothed. However, with the reconstructed data, users can see the variation of sensed data and should request the source node to retransmit the original data only if they need to confirm the exact value of data. The quantization step size is increased in proportion to the strength of congestion and the fineness (fidelity) of the reconstructed data decreases in inverse proportion to the quantization step size. Therefore, the fineness of reconstructed data decreases in inverse proportion to the congestion strength.

To reduce the packet drop and guarantee the fairness, ACT controls the forwarding rate and the generating rate together. When a node detects the congestion situation, the node notifies its child nodes to reduce the out rate. Child nodes compute the out rate for itself and its child node by dividing the available out rate with the whole number of its child including itself and then also notify their child nodes the congestion with proper out rate. Child nodes then apply the computed rate to the ARC and APC.

**Figure 12.**
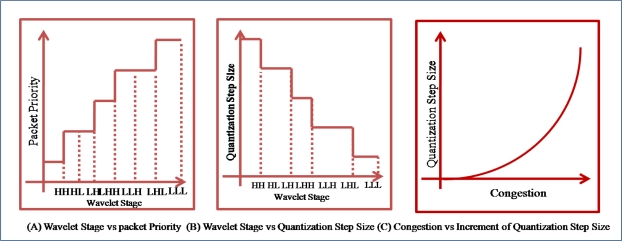
Priority and quantization.

The ACT assigns a priority to a packet based on the result of the DWT. The DWT divides data into four groups with different frequencies. Usually, a high-frequency group has little energy and may be disregarded, while a low-frequency group has considerable energy and should not be disregarded. Therefore, groups with a low frequency should be assigned a high priority in order to prevent a packet drop during data transmission, as shown in Figure 12-(A). The ACT uses the priority for the selection of a packet to drop and the selection of the quantization step size in ADPCM [Figure 12-(B)]. The bigger the quantization step size, the lesser the number of packets generated by RLC. Therefore, when congestion occurs in a network, the ACT should set up a big quantization step size. Moreover, the ACT increases the quantization step size in proportion to the congestion strength [Figure 12-(C)].

### 3.7. Energy

Transmission of data is one of the most energy expensive tasks that a node undertakes and using data compression to reduce the number of bits sent reduces energy for transmission. However, compression requires the computation, which also consumes the energy. Trading the computation for transmission can save the energy since typically on the order of 3,000 instructions can be executed for the energy cost required to transmit one bit over a distance of 100 m by radio [44]. Moreover, usually sensor nodes are deployed in the large area and thus packets are relayed through multiple sensor nodes. Therefore, the reduced amount of data with compression can reduce the energy consumption on the compressing node and the relaying nodes.

**Figure 13.**
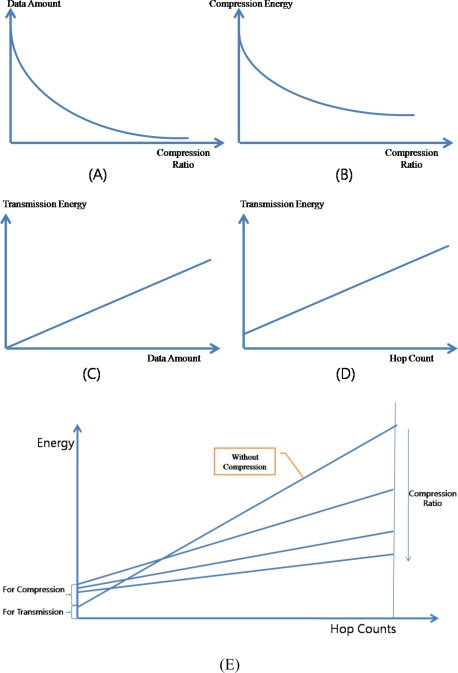
Data Compression and Energy Consumption.

Figure 13 presents the relation between the compression and the energy consumption. The amount of data generated by the compressor is in inverse proportion to the compression ratio [Figure 13-(A)] and the amount of energy consumed by the compressor is also in inverse proportion to the compression ratio [Figure 13-(B)]. In the ACT, workloads of DWT and ADPCM are constant regardless of the data, however the workload of RLC is in inverse proportion to the difference among data. Therefore, there is the minimum level of the consumed energy in the ACT. The amount of energy consumed by the packet transmission is in proportion to the amount of data transferring [Figure 13-(C)] and to the hop count which a packet is relayed [Figure 13-(D)]. As a consequence, ACT can reduce the total consumed energy by reducing the amount of data to send [Figure 13-(E)].

## 4. Performance

### 4.1. Experimental Setup

In order to evaluate the performance of the proposed ACT, we simulated CODA, CODA with compression at the source node (CODA-Comp), CRRT and the ACT together by using TOSSIM, which is a simulator for TinyOS. TinyOS is an operating system developed for event-based sensor networks at UC Berkeley. TOSSIM provides a simulation environment that simulates a real sensor network with TinyOS. An application in TinyOS consists of components from each network layer and hardware. The application running on TOSSIM can be run on real sensor nodes such as a micaz or a telos. Thus, we can say that our implemented simulation reflects the real world. Table 1 lists the specifications of simulation environment and Table 2 lists the specifications of the simulated sensor mote. Hundred sensor nodes sensed data at a random sensing interval between 100 ms to 1,000 ms and transferred the data to the sink node with a packet containing 10 data samples. We selected three performance matrixes: efficiency, fairness, QoD (quality of data), and energy [7,13].

### 4.2. Network Efficiency

The network efficiency measures the efficiency of packet transmission in a sensor node. If a packet is successfully delivered from a source node to a sink node, the efficiency is perfect (100%). However, if a packet is dropped somewhere in a network during transmission, the transmissions from the source node to the relaying node that dropped the packet become unnecessary. If many packets are dropped due to congestion, the network efficiency decreases. The network efficiency is related directly to energy because unnecessary packet transmissions unnecessarily dissipate a large amount of energy, which is a very critical resource.

**Figure 14.**
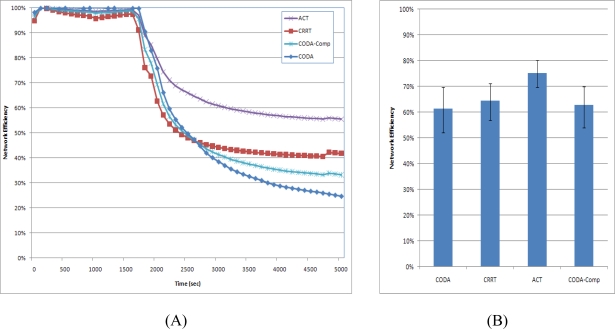
Network Efficiency.

Figure 14 shows the network efficiency of the CODA, CODA-Comp, CRRT and ACT. The total amount of sensed data per second slightly exceeded the available bandwidth at the beginning of the simulation, and hence, the efficiency of the CODA slightly decreased. During congestion, the CODA process drops overflowed packets and controls one sensor node among child nodes and sets it to the sleep state for some time. Therefore, the network efficiency decreased due to the overflowed packets in the congested nodes. As the back pressure (making one child node sleep) propagated slowly, the packet overflow decreased, and as a result, the network efficiency increased. However, as sensor nodes generated more packets and thus the network congestion became serious, more packets were dropped. As a consequence, the network efficiency drastically decreased. In the case of CODA-Comp, the packet is compressed before sending at the source node and thus less congestion is occurred. Therefore, CODA-Comp shows higher network efficiency than CODA. CRRT shows higher network efficiency than CODA-Comp. CRRT optimized the retransmission at the hop-by-hop communication and thus less packets were lost and thus the network efficiency was higher than CODA.

On the other hand, the ACT exhibits high efficiency at the beginning of simulation, because it reduces the generated packets with the APC. Although the total number of sensed data packets is increased and network congestion becomes serious, the ACT exhibits high network efficiency. Moreover, the network efficiency of CODA decreases continuously, while that of the ACT increases with time. With CODA, the back pressure is propagated slowly, and the packet flows greatly fluctuate in case of congestion. Therefore, the packet overflows increase, which leads to a decrease in the network efficiency. On the other hand, the ACT controls the quantization step size in proportion to the congestion state, and thus, the total number of packets in the network is decreased. Moreover, it propagates the back pressure fast with adaptive queue operation. As a consequence, its network efficiency is increases slowly. ACT also shows highest value at the average network efficiency [Figure 14-(B)]. The confidence interval was 95%.

### 4.3. Fairness

Fairness measures the packet reception ratio from each sensor node at the sink node. Fairness decreases if the sink node receives more packets from some sensor nodes than other sensor nodes and increases if the sink node receives packets from every node fairly. In the simulation, the fairness might be very low because sensor nodes selected the sensing interval randomly. If a sensor node selects a long sensing interval, it will generate packets at a low rate, and thus, less number of packets will be transferred to the sink node, while a sensor node with a short sensing interval will generate packets at a high rate. Moreover, a large number of packets from sensor nodes with a short sensing interval may cause congestion, and the packets from such sensor nodes will also be dropped. Therefore, the fairness will considerably decrease in case of heavy congestion.

**Figure 15.**
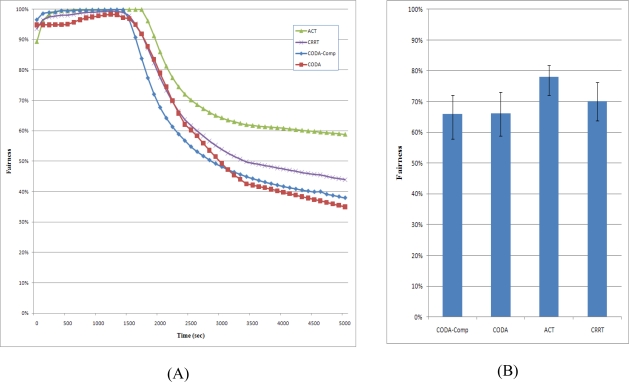
Fairness.

Figure 15 shows the fairness of CODA, CODA-Comp, CRRT, and ACT strategy. As congestion becomes serious, the fairness drastically decreases because the CODA process sets one child node to the sleep state for some time, which leads to unbalanced packet transmission. A sleeping node can send neither the packets generated by it nor the packets received from its child nodes. Moreover, the packet flows considerably fluctuate because the back pressure is propagated slowly. Therefore, the network fairness decreased drastically. The fairness of CODA-Comp also decreased drastically showing similar shape with CODA because CODA-Comp only applies the compression at the source node. CRRT shows higher fairness than CODA because CRRT reduces the collision among the nodes at the MAC layer. Actually the CRRT has the end-to-end retransmission technique for reliability however we turned off the end-to-end retransmission in the simulation. If the end-to-end retransmission is on, the fairness would be perfect.

On the other hand, the ACT exhibits higher fairness than the other strategies does because it simultaneously uses the back pressure and up pressure. It attempts to reduce the number of packets in the network with the help of the ARC. Further, sensor nodes with a short sensing interval will generate more packets that will in turn occupy more space in the queues of relaying nodes. Therefore, the ARC of the ACT attempts to reduce the number of these packets in the congested node. Moreover, with the ACT, all child nodes of the congested node increase the packet transmission interval, and thus, the ACT can achieve higher fairness than other schemes can.

### 4.4. QoD

**Figure 16.**
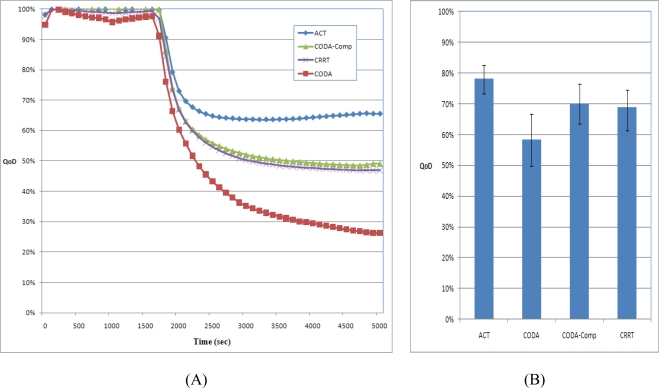
QoD.

QoD is defined as the ratio of the amount of data obtained by the sink node to the amount of data sensed by sensor nodes. The entire sensed data should arrive at the sink node and used by user applications. During congestion, the network should make the best effort to deliver as much data to the sink node as possible. Figure 16 shows the QoD of the CODA, CODA-Comp, CRRT, and ACT. As shown in Figure 14 and mentioned in Section 4.2, CODA exhibits very low network efficiency and therefore has a very low QoD. The network efficiency and the QoD of CODA should be similar because CODA does not use any compression technique. CODA-Comp and CRRT showed similar result at the network efficiency however CODA-Comp shows higher QoD in Figure 16 because with CODA-Comp at the sink received packets are decompressed and could reconstruct much more data than CRRT. In contrast, the ACT has considerably higher QoD than other schemes because the ACT uses compression techniques not only at the source node but also at the relaying node. When the network congestion occurs, ACT tries to compress the packets from the child nodes and thus the less amounts of packets were dropped. With a compression technique, a packet can contain more data than a packet without such a technique. This is the reason why compression techniques are used widely in several networks.

### 4.5. Energy

**Figure 17.**
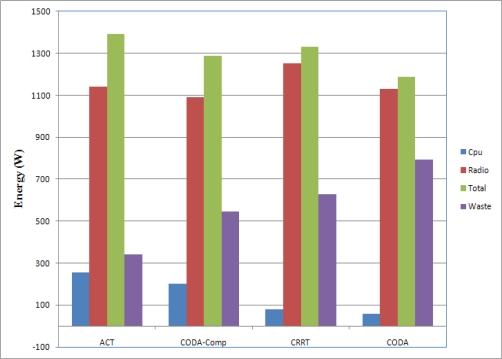
Energy Consumption.

Sensor nodes are usually powered by batteries and thus have very limited energy, therefore maximizing the network lifetime is the main objective of the majority of WSN research works. With data compression, the traffic is reduced and energy consumption is also reduced and the network lifetime can be extended. However ACT is composed by many compression techniques which could be expensive in terms of energy and thus energy efficiency should be compared. Figure 17 shows the comparison of energy consumption. The energy consumption by the CPU and the Radio (Network Interface Card) was estimated. CPU was used by the sensing component, compression component, and transmission component. The Radio consumption is the summation of energies consumed by the sending and receiving. If a packet is dropped somewhere in a network during transmission, the transmissions from the source node to the relaying node that dropped the packet become unnecessary and thus the energy which was consumed by the radio is wasted (“Waste” in the figure). CODA shows minimum amount of total energy consumption. With CODA, there is not much computation and thus the energy consumption by the CPU is very smaller than other schemes. However, CODA showed very poor network efficiency in Figure 14, which means lots of packet drop, and thus lots of energy was wasted. CODA with compression consumed less energy on the Radio and more energy on the CPU. Therefore the total amount of consumed energy is more than CODA whereas the amount of wasted energy is less than the CODA. CRRT stays in middle stage between CODA and CODA-Comp. ACT shows highest energy consumption than other scheme. With ACT, CPU consumed lots of energy due to the compression task, however the entire network efficiency was higher than other schemes. Therefore the wasted energy was less than other scheme.

## 5. Conclusions

Recent advances in MEMS and microprocessor and wireless communication technologies have enabled the deployment of large-scale sensor networks, where thousands or even tens of thousands of small sensors are distributed over a vast area in order to collect sensing data. A large amount of data from a large number of sensor nodes can provide very important information to the users; however, a very large amount of data might cause large-scale congestion in a network.

Conventional congestion control protocols usually use a back-pressure scheme that reduces the congestion by reducing the transfer rate of the child nodes of the congested node and by decreasing the packet generation rate of the sensor node that causes heavy traffic. However, packet drops may still occur because the back pressure is propagated slowly due to the collision among sensor nodes in case of serious congestion.

In this paper, we proposed a new congestion control technique, named ACT, based on an adaptive compression scheme for packet reduction in case of congestion. Compression techniques used in the ACT are DWT, ADPCM, and RLC. ACT first transforms the data from the time domain to the frequency domain, reduces the range of the data with the help of ADPCM, and then reduces the number of packets by using RLC before transferring the data to the source node. It then introduces the DWT for priority-based congestion control because the DWT classifies the data into four groups with different frequencies. Subsequently, it assigns priorities to these data groups in an inverse proportion to the respective frequencies of the data groups and defines the quantization step size of ADPCM in an inverse proportion to the priorities. RLC generates a smaller number of packets for a data group with a low priority. In the relaying node, the ACT reduces the number of packets by increasing the quantization step size of ADPCM in case of congestion. Moreover, in order to facilitate the back pressure, the queue is controlled adaptively according to the congestion state. We experimentally demonstrated that the ACT increases the network efficiency and guarantees fairness to sensor nodes, as compared with the existing methods and that it exhibits very high ratio of the overall available data in the sink.

## Figures and Tables

**Table 1.** Specifications of simulation environment.

**Parameter**	**Value**

Radio Range	100 m
Number of sensor nodes	100
Simulation Time	5,000 sec
Max Network Bandwidth	40 packets/sec
Sampling Interval	10∼1,000 ms
Queue size on a sensor node	64
Max transmission interval	255 ms
Event Change	2,000 sec

**Table 2.** Specifications of simulated mote.

MCU	ATMEGA 128 L 8 MHz

Memory	4 K RAM/128 K FLASH

RF Transceiver	Chipcon CC2420
	IEEE 802.15.4/ZigBee compliant
	2.4 GHz Frequency band
	250 Kbps Transmission data rate
	−24 dBm to 0 dBm RF power
	20 m to 30 m Indoor range
